# Supplementary material for: Getting More Out of Biomedical Documents with GATE's Full Lifecycle Open Source Text Analytics
Source: PLoS Comput Biol. 2013 Feb 7;9(2):e1002854. doi: 10.1371/journal.pcbi.1002854 (PMC3567135; doi:10.1371/journal.pcbi.1002854)
Supplement: Dataset S1 — GATE software. Dataset S1 bundled with this paper contains a distribution of GATE (or see http://gate.ac.uk/download/). (TGZ) [file pcbi.1002854.s001.tgz › plos-gate/plugins/Tagger_MetaMap/doc/gate/metamap/MetaMapPR.html]

MetaMapPR


---


|  |  |  |
| --- | --- | --- |
| **Overview** \| **Package** \| **Class** \| **Use** \| **Tree** \| **Deprecated** \| **Index** \| **Help** | |  |
| PREV CLASS   **NEXT CLASS** | **FRAMES**    **NO FRAMES**     **All Classes** |  |
| SUMMARY: NESTED \| FIELD \| CONSTR \| METHOD | DETAIL: FIELD \| CONSTR \| METHOD |  |


---


## gate.metamap Class MetaMapPR

```
java.lang.Object
  gate.util.AbstractFeatureBearer
      gate.creole.AbstractResource
          gate.creole.AbstractProcessingResource
              gate.creole.AbstractLanguageAnalyser
                  gate.metamap.MetaMapPR
```

**All Implemented Interfaces:**: gate.creole.ANNIEConstants, gate.Executable, gate.LanguageAnalyser, gate.ProcessingResource, gate.Resource, gate.util.FeatureBearer, gate.util.NameBearer, java.io.Serializable

---

``` @CreoleResource(name="MetaMap Annotator", helpURL="http://gate.ac.uk/userguide/sec:misc-creole:metamap", comment="This plugin uses the MetaMap Java API to send GATE document content to MetaMap skrmedpostctl server and PrologBeans mmserver instances running on the given machine/port") public class MetaMapPR extends gate.creole.AbstractLanguageAnalyser implements gate.ProcessingResource, java.io.Serializable ```

This class is the implementation of the resource METAMAP.

**See Also:**: Serialized Form

---

**Nested Class Summary**

**Nested classes/interfaces inherited from class gate.creole.AbstractProcessingResource**

`gate.creole.AbstractProcessingResource.InternalStatusListener, gate.creole.AbstractProcessingResource.IntervalProgressListener`


**Field Summary**

**Fields inherited from class gate.creole.AbstractLanguageAnalyser**

`corpus, document`

**Fields inherited from class gate.creole.AbstractProcessingResource**

`interrupted`

**Fields inherited from class gate.creole.AbstractResource**

`name`

**Fields inherited from class gate.util.AbstractFeatureBearer**

`features`

**Fields inherited from interface gate.creole.ANNIEConstants**

`ANNOTATION_COREF_FEATURE_NAME, DATE_ANNOTATION_TYPE, DATE_POSTED_ANNOTATION_TYPE, DEFAULT_FILE, DOCUMENT_COREF_FEATURE_NAME, JOB_ID_ANNOTATION_TYPE, LOCATION_ANNOTATION_TYPE, LOOKUP_ANNOTATION_TYPE, LOOKUP_CLASS_FEATURE_NAME, LOOKUP_INSTANCE_FEATURE_NAME, LOOKUP_LANGUAGE_FEATURE_NAME, LOOKUP_MAJOR_TYPE_FEATURE_NAME, LOOKUP_MINOR_TYPE_FEATURE_NAME, LOOKUP_ONTOLOGY_FEATURE_NAME, MONEY_ANNOTATION_TYPE, ORGANIZATION_ANNOTATION_TYPE, PERSON_ANNOTATION_TYPE, PERSON_GENDER_FEATURE_NAME, PLUGIN_DIR, PR_NAMES, SENTENCE_ANNOTATION_TYPE, SPACE_TOKEN_ANNOTATION_TYPE, TOKEN_ANNOTATION_TYPE, TOKEN_CATEGORY_FEATURE_NAME, TOKEN_KIND_FEATURE_NAME, TOKEN_LENGTH_FEATURE_NAME, TOKEN_ORTH_FEATURE_NAME, TOKEN_STRING_FEATURE_NAME`


**Constructor Summary**

`MetaMapPR()`


| **Method Summary** | |
| --- | --- |
| `void` | `doCoreferenceAnnots(java.util.HashMap<java.lang.Integer,java.util.ArrayList<java.lang.Integer>> termMapById, gate.AnnotationSet inputAS, gate.AnnotationSet outputAS)` |
| `void` | `execute()` |
| `java.lang.String` | `filterNonAscii(java.lang.String text)` |
| `java.lang.Boolean` | `getAnnotateNegEx()` |
| `java.lang.Boolean` | `getAnnotatePhrases()` |
| `java.lang.String` | `getInputASName()` |
| `java.lang.String` | `getInputASTypeFeature()` |
| `java.util.ArrayList<java.lang.String>` | `getInputASTypes()` |
| `java.lang.Integer` | `getLinebreakCount()` |
| `java.lang.String` | `getMetaMapOptions()` |
| `java.lang.String` | `getOutputASName()` |
| `java.lang.String` | `getOutputASType()` |
| `OutputMode` | `getOutputMode()` |
| `TaggerMode` | `getTaggerMode()` |
| `gate.Resource` | `init()` |
| `java.lang.String` | `normalizeString(java.lang.String str)` |
| `void` | `processCandidates(gov.nih.nlm.nls.metamap.PCM pcm, java.util.List<gov.nih.nlm.nls.metamap.Negation> negList, java.lang.Long lngInitialOffset, java.lang.Long lngEndOffset)` |
| `void` | `processEvents(java.util.List<gov.nih.nlm.nls.metamap.Ev> eVList, java.util.List<gov.nih.nlm.nls.metamap.Negation> negList, java.lang.String type, java.lang.Long lngInitialOffset, java.lang.Long lngEndOffset)` |
| `void` | `processMappings(gov.nih.nlm.nls.metamap.PCM pcm, java.util.List<gov.nih.nlm.nls.metamap.Negation> negList, java.lang.Long lngInitialOffset, java.lang.Long lngEndOffset)` |
| `void` | `processPhrase(gov.nih.nlm.nls.metamap.PCM pcm, java.lang.Long lngInitialOffset)` |
| `void` | `processUtterances(gov.nih.nlm.nls.metamap.Result result, java.lang.Long lngInitialOffset, java.lang.Long lngEndOffset)` |
| `void` | `processWithMetaMap(gov.nih.nlm.nls.metamap.MetaMapApi api, java.lang.String text, java.lang.Long lngInitialOffset, java.lang.Long lngEndOffset)` |
| `void` | `setAnnotateNegEx(java.lang.Boolean annotateNegEx)` |
| `void` | `setAnnotatePhrases(java.lang.Boolean annotatePhrases)` |
| `void` | `setInputASName(java.lang.String inputASName)` |
| `void` | `setInputASTypeFeature(java.lang.String inputASTypeFeature)` |
| `void` | `setInputASTypes(java.util.ArrayList<java.lang.String> inputASTypes)` |
| `void` | `setLinebreakCount(java.lang.Integer linebreakCount)` |
| `void` | `setMetaMapOptions(java.lang.String metaMapOptions)` |
| `void` | `setOutputASName(java.lang.String outputASName)` |
| `void` | `setOutputASType(java.lang.String outputASType)` |
| `void` | `setOutputMode(OutputMode outputMode)` |
| `void` | `setTaggerMode(TaggerMode taggerMode)` |
| `gov.nih.nlm.nls.metamap.MetaMapApi` | `setUpApi()` |

**Methods inherited from class gate.creole.AbstractLanguageAnalyser**

`getCorpus, getDocument, setCorpus, setDocument`

**Methods inherited from class gate.creole.AbstractProcessingResource**

`addProgressListener, addStatusListener, cleanup, fireProcessFinished, fireProgressChanged, fireStatusChanged, interrupt, isInterrupted, reInit, removeProgressListener, removeStatusListener`

**Methods inherited from class gate.creole.AbstractResource**

`checkParameterValues, getBeanInfo, getName, getParameterValue, getParameterValue, removeResourceListeners, setName, setParameterValue, setParameterValue, setParameterValues, setParameterValues, setResourceListeners`

**Methods inherited from class gate.util.AbstractFeatureBearer**

`getFeatures, setFeatures`

**Methods inherited from class java.lang.Object**

`clone, equals, finalize, getClass, hashCode, notify, notifyAll, toString, wait, wait, wait`

**Methods inherited from interface gate.ProcessingResource**

`reInit`

**Methods inherited from interface gate.Resource**

`cleanup, getParameterValue, setParameterValue, setParameterValues`

**Methods inherited from interface gate.util.FeatureBearer**

`getFeatures, setFeatures`

**Methods inherited from interface gate.util.NameBearer**

`getName, setName`

**Methods inherited from interface gate.Executable**

`interrupt, isInterrupted`

**Constructor Detail**

### MetaMapPR

```
public MetaMapPR()
```


**Method Detail**

### init

```
public gate.Resource init()
                   throws gate.creole.ResourceInstantiationException
```

:   **Specified by:**: `init` in interface `gate.Resource` **Overrides:**: `init` in class `gate.creole.AbstractProcessingResource`
:   **Throws:**: `gate.creole.ResourceInstantiationException`

---


### execute

```
public void execute()
             throws gate.creole.ExecutionException
```

:   **Specified by:**: `execute` in interface `gate.Executable` **Overrides:**: `execute` in class `gate.creole.AbstractProcessingResource`
:   **Throws:**: `gate.creole.ExecutionException`

---


### doCoreferenceAnnots

```
public void doCoreferenceAnnots(java.util.HashMap<java.lang.Integer,java.util.ArrayList<java.lang.Integer>> termMapById,
                                gate.AnnotationSet inputAS,
                                gate.AnnotationSet outputAS)
                         throws gate.creole.ExecutionException
```

:   **Parameters:**: `termMapById` - HashMap containing first occurrence of each of term: `inputAS` - input AnnotationSet: `outputAS` - output AnnotationSet **Throws:**: `gate.creole.ExecutionException`

---


### setUpApi

```
public gov.nih.nlm.nls.metamap.MetaMapApi setUpApi()
                                            throws java.lang.Exception
```

:   **Returns:**: MetaMapApi instance **Throws:**: `java.lang.Exception`

---


### processPhrase

```
public void processPhrase(gov.nih.nlm.nls.metamap.PCM pcm,
                          java.lang.Long lngInitialOffset)
                   throws java.lang.Exception
```

:   **Parameters:**: `pcm` - - phrase chunk returned by MetaMap: `lngInitialOffset` - - offset start of annotation relative to prior text **Throws:**: `java.lang.Exception`

---


### processEvents

```
public void processEvents(java.util.List<gov.nih.nlm.nls.metamap.Ev> eVList,
                          java.util.List<gov.nih.nlm.nls.metamap.Negation> negList,
                          java.lang.String type,
                          java.lang.Long lngInitialOffset,
                          java.lang.Long lngEndOffset)
                   throws java.lang.Exception
```

:   **Parameters:**: `eVList` - - list of events returned by MetaMap: `negList` - - list of negated terms returned by MetaMap: `type` - - Candidate or Mapping: `lngInitialOffset` - - offset start of annotation relative to prior text: `lngEndOffset` - - use existing annotation end if annotating feature inside annotation **Throws:**: `java.lang.Exception`

---


### processMappings

```
public void processMappings(gov.nih.nlm.nls.metamap.PCM pcm,
                            java.util.List<gov.nih.nlm.nls.metamap.Negation> negList,
                            java.lang.Long lngInitialOffset,
                            java.lang.Long lngEndOffset)
                     throws java.lang.Exception
```

:   **Parameters:**: `pcm` - - phrase chunk returned by MetaMap: `negList` - - list of negated terms returned by MetaMap: `lngInitialOffset` - - offset start of annotation relative to prior text: `lngEndOffset` - - use existing annotation end if annotating feature inside annotation **Throws:**: `java.lang.Exception`

---


### processCandidates

```
public void processCandidates(gov.nih.nlm.nls.metamap.PCM pcm,
                              java.util.List<gov.nih.nlm.nls.metamap.Negation> negList,
                              java.lang.Long lngInitialOffset,
                              java.lang.Long lngEndOffset)
                       throws java.lang.Exception
```

:   **Parameters:**: `pcm` - - phrase chunk returned by MetaMap: `negList` - - list of negated terms returned by MetaMap: `lngInitialOffset` - - offset start of annotation relative to prior text: `lngEndOffset` - - use existing annotation end if annotating feature inside annotation **Throws:**: `java.lang.Exception`

---


### processUtterances

```
public void processUtterances(gov.nih.nlm.nls.metamap.Result result,
                              java.lang.Long lngInitialOffset,
                              java.lang.Long lngEndOffset)
                       throws java.lang.Exception
```

:   **Parameters:**: `result` - - result returned by MetaMap: `lngInitialOffset` - - offset start of annotation relative to prior text: `lngEndOffset` - - use existing annotation end if annotating feature inside annotation **Throws:**: `java.lang.Exception`

---


### processWithMetaMap

```
public void processWithMetaMap(gov.nih.nlm.nls.metamap.MetaMapApi api,
                               java.lang.String text,
                               java.lang.Long lngInitialOffset,
                               java.lang.Long lngEndOffset)
                        throws java.lang.Exception
```

:   **Parameters:**: `api` - - MetaMapApi instance: `text` - - text to be annotated: `lngInitialOffset` - - offset start of annotation relative to prior text: `lngEndOffset` - - use existing annotation end if annotating feature inside annotation **Throws:**: `java.lang.Exception`

---


### normalizeString

```
public java.lang.String normalizeString(java.lang.String str)
                                 throws java.lang.ClassNotFoundException
```

:   **Parameters:**: `str` - **Returns:**: Normalized version of str with accented characters replaced by unaccented version and with diacritics removed. E.g. Ö -> O **Throws:**: `java.lang.ClassNotFoundException`

---


### filterNonAscii

```
public java.lang.String filterNonAscii(java.lang.String text)
                                throws java.io.UnsupportedEncodingException
```

:   **Parameters:**: `text` - **Returns:**: ASCII encoding of text with non ASCII characters replaced by ? **Throws:**: `java.io.UnsupportedEncodingException`

---


### setInputASName

```
@Optional
@RunTime
@CreoleParameter(comment="Input Annotation Set Name")
public void setInputASName(java.lang.String inputASName)
```

---


### getInputASName

```
public java.lang.String getInputASName()
```

---


### setOutputASName

```
@Optional
@RunTime
@CreoleParameter(comment="Output Annotation Set Name")
public void setOutputASName(java.lang.String outputASName)
```

---


### getOutputASName

```
public java.lang.String getOutputASName()
```

---


### setOutputASType

```
@RunTime
@CreoleParameter(defaultValue="MetaMap",
                 comment="Name for the MetaMap Annotation types")
public void setOutputASType(java.lang.String outputASType)
```

---


### getOutputASType

```
public java.lang.String getOutputASType()
```

---


### setInputASTypes

```
@Optional
@RunTime
@CreoleParameter(comment="If set, only send the content of the given Annotations in the input Annotation Set to MetaMap")
public void setInputASTypes(java.util.ArrayList<java.lang.String> inputASTypes)
```

---


### getInputASTypes

```
public java.util.ArrayList<java.lang.String> getInputASTypes()
```

---


### setInputASTypeFeature

```
@Optional
@RunTime
@CreoleParameter(comment="If set, only send the content of the given feature from annotations within inputASTypes to MetaMap")
public void setInputASTypeFeature(java.lang.String inputASTypeFeature)
```

---


### getInputASTypeFeature

```
public java.lang.String getInputASTypeFeature()
```

---


### setOutputMode

```
@RunTime
@CreoleParameter(defaultValue="HighestMappingOnly",
                 comment="Output highest scoring final mapping, all mappings, all candidate terms, or all candidates and mappings")
public void setOutputMode(OutputMode outputMode)
```

---


### getOutputMode

```
public OutputMode getOutputMode()
```

---


### setAnnotatePhrases

```
@RunTime
@CreoleParameter(defaultValue="false",
                 comment="Output MetaMap phrase-level annotations?")
public void setAnnotatePhrases(java.lang.Boolean annotatePhrases)
```

---


### getAnnotatePhrases

```
public java.lang.Boolean getAnnotatePhrases()
```

---


### setMetaMapOptions

```
@Optional
@RunTime
@CreoleParameter(defaultValue="-Xdt",
                 comment="MetaMap runtime options")
public void setMetaMapOptions(java.lang.String metaMapOptions)
```

---


### getMetaMapOptions

```
public java.lang.String getMetaMapOptions()
```

---


### setAnnotateNegEx

```
@RunTime
@CreoleParameter(defaultValue="false",
                 comment="Output NegEx negation annotations and features?")
public void setAnnotateNegEx(java.lang.Boolean annotateNegEx)
```

---


### getAnnotateNegEx

```
public java.lang.Boolean getAnnotateNegEx()
```

---


### setLinebreakCount

```
@RunTime
@CreoleParameter(defaultValue="2",
                 comment="Number of linebreak/whitespace characters between paragraphs")
public void setLinebreakCount(java.lang.Integer linebreakCount)
```

---


### getLinebreakCount

```
public java.lang.Integer getLinebreakCount()
```

---


### setTaggerMode

```
@RunTime
@CreoleParameter(defaultValue="CoReference",
                 comment="Map first instance of a term only, first instance plus coreferences, or all instances independently")
public void setTaggerMode(TaggerMode taggerMode)
```

---


### getTaggerMode

```
public TaggerMode getTaggerMode()
```


---


|  |  |  |
| --- | --- | --- |
| **Overview** \| **Package** \| **Class** \| **Use** \| **Tree** \| **Deprecated** \| **Index** \| **Help** | |  |
| PREV CLASS   **NEXT CLASS** | **FRAMES**    **NO FRAMES**     **All Classes** |  |
| SUMMARY: NESTED \| FIELD \| CONSTR \| METHOD | DETAIL: FIELD \| CONSTR \| METHOD |  |


---
